# Differential Inputs to the Perisomatic and Distal-Dendritic Compartments of VIP-Positive Neurons in Layer 2/3 of the Mouse Barrel Cortex

**DOI:** 10.3389/fnana.2016.00124

**Published:** 2016-12-20

**Authors:** Jaerin Sohn, Shinichiro Okamoto, Naoya Kataoka, Takeshi Kaneko, Kazuhiro Nakamura, Hiroyuki Hioki

**Affiliations:** ^1^Department of Morphological Brain Science, Graduate School of Medicine, Kyoto UniversityKyoto, Japan; ^2^Division of Cerebral Circuitry, National Institute for Physiological SciencesOkazaki, Japan; ^3^Department of Integrative Physiology, Nagoya University Graduate School of MedicineNagoya, Japan; ^4^Precursory Research for Embryonic Science and Technology (PRESTO), Japan Science and Technology Agency (JST)Kawaguchi, Japan

**Keywords:** barrel cortex, confocal laser scanning microscopy, GABAergic neuron, somatodendritic compartment, vasoactive intestinal polypeptide

## Abstract

The recurrent network composed of excitatory and inhibitory neurons is fundamental to neocortical function. Inhibitory neurons in the mammalian neocortex are molecularly diverse, and individual cell types play unique functional roles in the neocortical microcircuit. Recently, vasoactive intestinal polypeptide-positive (VIP+) neurons, comprising a subclass of inhibitory neurons, have attracted particular attention because they can disinhibit pyramidal cells through inhibition of other types of inhibitory neurons, such as parvalbumin- (PV+) and somatostatin-positive (SOM+) inhibitory neurons, promoting sensory information processing. Although VIP+ neurons have been reported to receive synaptic inputs from PV+ and SOM+ inhibitory neurons as well as from cortical and thalamic excitatory neurons, the somatodendritic localization of these synaptic inputs has yet to be elucidated at subcellular spatial resolution. In the present study, we visualized the somatodendritic membranes of layer (L) 2/3 VIP+ neurons by injecting a newly developed adeno-associated virus (AAV) vector into the barrel cortex of VIP-Cre knock-in mice, and we determined the extensive ramification of VIP+ neuron dendrites in the vertical orientation. After immunohistochemical labeling of presynaptic boutons and postsynaptic structures, confocal laser scanning microscopy revealed that the synaptic contacts were unevenly distributed throughout the perisomatic (<100 μm from the somata) and distal-dendritic compartments (≥100 μm) of VIP+ neurons. Both corticocortical and thalamocortical excitatory neurons preferentially targeted the distal-dendritic compartment of VIP+ neurons. On the other hand, SOM+ and PV+ inhibitory neurons preferentially targeted the distal-dendritic and perisomatic compartments of VIP+ neurons, respectively. Notably, VIP+ neurons had few reciprocal connections. These observations suggest different inhibitory effects of SOM+ and PV+ neuronal inputs on VIP+ neuron activity; inhibitory inputs from SOM+ neurons likely modulate excitatory inputs locally in dendrites, while PV+ neurons could efficiently interfere with action potential generation through innervation of the perisomatic domain of VIP+ neurons. The present study, which shows a precise configuration of site-specific inputs, provides a structural basis for the integration mechanism of synaptic inputs to VIP+ neurons.

## Introduction

In the neocortex, γ-aminobutyric acid-ergic (GABAergic) inhibitory neurons display a large diversity of dendritic and axonal morphologies, intrinsic firing properties and chemical characteristics (Markram et al., 2004; Ascoli et al., 2008; Kubota, 2014; Zeisel et al., 2015; Tasic et al., 2016). Parvalbumin- (PV+), somatostatin- (SOM+), and vasoactive intestinal polypeptide-positive (VIP+) neurons are the three major subclasses of GABAergic neurons (Xu et al., 2010; Rudy et al., 2011; Hioki et al., 2013). Individual GABAergic cell types play unique functional roles in the neocortical microcircuit (Gentet et al., 2012; Lee et al., 2012, 2013; Lovett-Barron et al., 2012; Wilson et al., 2012; Pi et al., 2013; Fu et al., 2014; Zhang et al., 2014), and they make reciprocal synaptic connections with each other (Isaacson and Scanziani, 2011; Pfeffer et al., 2013).

VIP+ neurons are mainly distributed in layer (L) 2/3 of the rodent neocortex (Connor and Peters, 1984; Bayraktar et al., 2000; Xu et al., 2010; Rudy et al., 2011; Prönneke et al., 2015), and L2/3 VIP+ neurons are different from those in deeper layers in terms of morphological, electrophysiological and molecular features (Prönneke et al., 2015; Tasic et al., 2016). Most L2/3 VIP+ neurons extend their dendrites bidirectionally in the vertical orientation, morphologically characterized as bipolar/modified bipolar/bitufted cells, and send axon fibers vertically and translaminarly across L1–6 (Connor and Peters, 1984; Kawaguchi and Kubota, 1996; Bayraktar et al., 2000; Prönneke et al., 2015). Activation of VIP+ neurons potentiates the excitability of pyramidal cells through inhibition of other types of inhibitory neurons innervating pyramidal cells, which have been found mainly in L2/3 of sensory cortices (Lee et al., 2013; Pfeffer et al., 2013; Pi et al., 2013; Fu et al., 2014; Zhang et al., 2014). Hence, the activity regulation of L2/3 VIP+ neurons is crucial for the gain control of pyramidal cell response to sensory inputs in sensory cortices (Kepecs and Fishell, 2014; Pfeffer, 2014).

The impacts of excitatory and inhibitory inputs on the activity of VIP+ neurons have been investigated with electrophysiological and optogenetic techniques at cellular spatial resolution by measuring the strength of cell-to-cell connections using somatic recording (Porter et al., 1998; Rozov et al., 2001; Lee et al., 2013; Pfeffer et al., 2013; Pi et al., 2013; Zhang et al., 2014). However, anatomical information on the subcellular localizations of synaptic input sites on VIP+ neurons is unavailable due to limited spatial resolution. The proximal and distal dendritic portions of GABAergic neurons exhibit different properties in action potential backpropagation and calcium dynamics (Goldberg et al., 2003), indicating that synaptic inputs to the different somatodendritic compartments have different weights on computation within postsynaptic domains. The knowledge of the precise configuration of synaptic inputs to the somatodendritic structures in VIP+ neurons, therefore, should be indispensable for further understanding of the activity regulation of VIP+ neurons, which affects the excitability of pyramidal cells.

In the present study, we morphologically analyzed the spatial pattern of synaptic inputs to VIP+ neurons at subcellular spatial resolution in L2/3 of the mouse primary somatosensory cortex barrel field (S1BF). The somatodendritic plasma membranes of VIP+ neurons were selectively visualized with tagged green fluorescent protein (GFP) by transduction with a recombinant adeno-associated virus (AAV) vector in VIP-Cre knock-in mice, and excitatory and inhibitory input sites were immunohistochemically visualized. Imaging studies of the visualized samples by confocal laser scanning microscopy were performed to analyze the distribution of synaptic input sites on the somatodendritic membranes of VIP+ neurons. Finally, we assessed positional preferences of the synaptic inputs on VIP+ somatodendritic compartments.

## Materials and Methods

### Animals

All animal care and use were in accordance with the National Institutes of Health Guide for the Care and Use of Laboratory Animals, and experiments were approved by the Committee for Animal Care and Use (MedKyo 15012, MedKyo 16573) and the Committee for Recombinant DNA Study (120093, 141008) of Kyoto University. Two to three months old adult male C57BL/6J mice (Japan SLC, Hamamatsu, Japan) and Vip^tm1(cre)Zjh^/J (VIP-Cre) mice (The Jackson Laboratory, Bar Harbor, ME, USA; stock number 010908; Taniguchi et al., 2011) were used in the present study. All efforts were made to minimize animal suffering and the number of animals used.

### Preparation of AAV2/1-SynTetOff-FLEX-FGL

**Figure 1:**
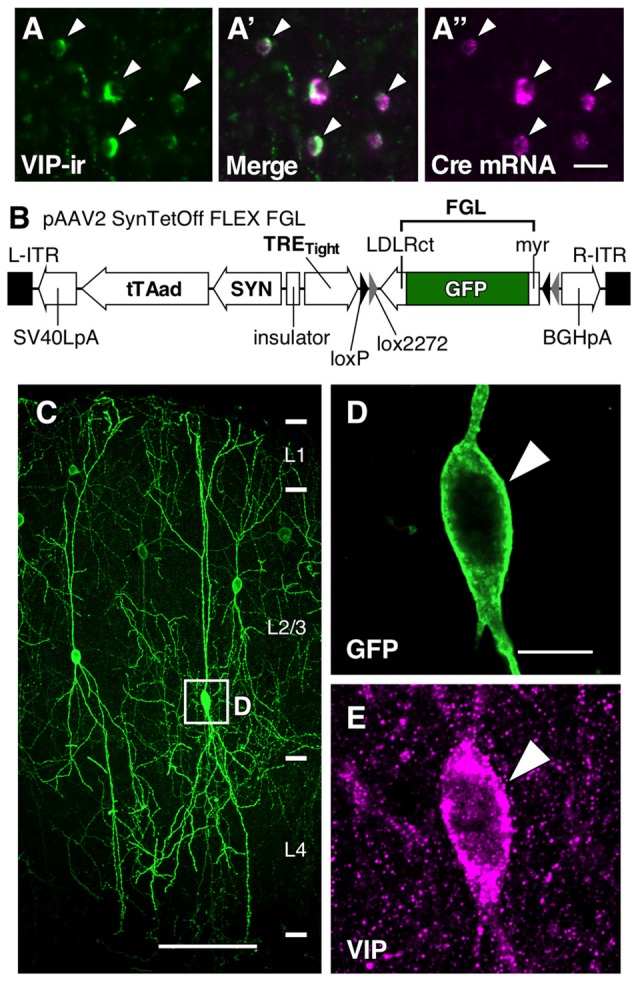
**Somatodendritic visualization of vasoactive intestinal polypeptide positive (VIP+) neurons. (A–A″)** Specific expression of Cre mRNA in VIP-Cre knock-in mice. In the primary somatosensory cortex barrel field (S1BF) of VIP-Cre knock-in mice, 98.6 ± 0.4% (mean ± SD) of VIP-immunoreactive (VIP-ir) cells expressed Cre mRNA, and inversely, 98.2 ± 0.8% of Cre-expressing cells showed immunoreactivity for VIP. Arrowhead indicates a double-labeled neuron. Scale bar = 20 μm. **(B)** Construction of pAAV2-SynTetOff-FLEX-FGL. Both the myristoylation/palmitoylation site of the Fyn N-terminus (myr) and the C-terminus of the low-density lipoprotein receptor (LDLRct) were added to green fluorescent protein (GFP), referred to as FGL. FGL was previously reported to be distributed specifically on the somatodendritic membranes of neurons (Kameda et al., 2008, 2012). **(C–E)** Specific labeling of somatodendritic membranes of VIP+ neurons. One week after injection of AAV2/1-SynTetOff-FLEX-FGL into the S1BF of VIP-Cre knock-in mice, brain sections were double-immunostained for GFP and VIP. GFP was specifically expressed in VIP+ neurons (**E**; 99.0 ± 0.1% of GFP-positive cells were VIP-ir). The numerical data were acquired bilaterally from three mice (309 GFP-positive cells). Arrowheads indicate a GFP-expressing VIP+ neuron. Scale bars in **(C,D)** = 100 μm and 10 μm, respectively.

Previously reported with lentivirus vectors used to induce strong expression of the reporter protein in infected neurons (Hioki et al., 2009), the “single virus vector Tet-Off platform” was applied to an AAV vector. pAAV2-SynTetOff-FLEX-FGL was composed of a regulator and a response element separated by the chicken β-globin insulator (Kawashima et al., 2009) in a single AAV genome (Figure 1B). The regulator element expressed an improved version of a tetracycline-controlled transactivator (tTAad; Clontech, Palo Alto, CA, USA) under the control of human synapsin I (SYN) promoter (Hioki et al., 2007; nucleotides 1889−2289 in GenBank accession no. M55301.1), and the response element expressed a reporter protein under the control of a tetracycline response element (TRE_Tight_; Clontech) promoter, as used in lentivirus vectors (Hioki et al., 2009). myrGFP-LDLRct, referred to as FGL in the present study, was previously developed for specific labeling of the somatodendritic plasma membranes of neurons. FGL was composed of the myristoylation/palmitoylation site of Fyn N-terminus, GFP and the C-terminus of low density lipoprotein receptor (Kameda et al., 2008, 2012). The sequence of the flip-excision (FLEX) switch (Schnütgen et al., 2003) consisted of two pairs of *loxP* and *lox2272* sites in opposite orientations, and the FGL sequence was introduced between these pairs in the antisense orientation.

Virus particles were produced and purified as previously reported (Kataoka et al., 2014; Suzuki et al., 2015; Hamamoto et al., 2016). Briefly, pAAV2-SynTetOff-FLEX-FGL and helper plasmids (pXR1, University of North Carolina at Chapel Hill; pHelper, Stratagene, La Jolla, CA, USA) were cotransfected into HEK293T cells (RCB2202, Riken, Japan) with polyethylenimine (23966-2, Polysciences, Inc., Warrington, PA, USA). Virus particles were extracted by 3-time freeze-and-thaw process, purified from the crude lysate of the cells by ultracentrifugation with OptiPrep (Axis-Shield, Oslo, Norway), and then concentrated by ultrafiltration with Amicon Ultra-15 (NMWL 50K; Merck Millipore, Darmstadt, Germany). The virus titer (infectious units/ml, IFU/ml) was adjusted to 1.0 × 10^11^ IFU/ml with phosphate-buffered 0.9% (w/v) saline (PBS; pH 7.4), and the virus solution was stored in aliquots at −80°C until use for delivery to brain tissues.

### Virus Injection

Animals were deeply anesthetized with chloral hydrate (7 mg/10 g body weight) and mounted onto a stereotaxic apparatus. We injected 0.2 μl of the virus solution containing the vector AAV2/1-SynTetOff-FLEX-FGL into the S1BF of VIP-Cre mice (1.0 mm posterior to Bregma, 3.0 mm lateral to the midline, and 0.3–0.5 mm deep from the brain surface) by pressure through a glass micropipette attached to a Picospritzer III (Parker Hannifin Corporation, Cleveland, OH, USA). The animals were maintained under regular health checks for 1 week and then subjected to transcardial perfusion as described below.

### Fluorescence *in situ* Hybridization for Cre mRNA

Double labeling using fluorescence *in situ* hybridization for Cre and immunofluorescence staining for VIP was performed. Animals were deeply anesthetized with chloral hydrate (7 mg/10 g body weight) and perfused with 4% (w/v) formaldehyde in 0.1 M phosphate buffer (PB; pH 7.4), followed by postfixation in the same fixative at 4°C for 3 days. After cryoprotection with 30% (w/v) sucrose in PB, brain blocks were cut into 20-μm-thick coronal sections on a freezing microtome. The sense and antisense single-strand RNA probes for Cre (GenBank accession number: NC_005856.1) were synthesized with a digoxigenin (DIG) labeling kit (Roche Diagnostics, Mannheim, Germany).

The following hybridization procedure was performed as previously reported (Hioki et al., 2010, 2013; Ma et al., 2011; Sohn et al., 2014). Briefly, free-floating brain sections were hybridized at 60°C with 1 μg/ml cRNA probes in a hybridization buffer. After ribonuclease A treatment, the sections were incubated overnight with a mixture of 1:4000-diluted peroxidase-conjugated anti-DIG sheep antibody (11-093-274-910; Roche Diagnostics, Indianapolis, IN, USA) and 1:500-diluted anti-VIP rabbit serum (Table 1). The sections were then processed using a biotinylated tyramine-glucose oxidase (BT-GO) amplification method (Furuta et al., 2009; Kuramoto et al., 2009). First, sections were incubated in PB containing 0.31 mM BT, 3 μg/mL GO, 2 mg/mL β-D-glucose (Tokyo Chemical Industry America, Portland, OR, USA) and 2% bovine serum albumin. After washing, the sections were further incubated with a mixture of 5 μg/ml of Alexa Fluor 594-conjugated streptavidin (S-11227; Life Technologies, Carlsbad, CA, USA) and 5 μg/ml of Alexa Fluor 488-conjugated goat antibody to rabbit IgG (Table 1). After immunolabeling, the sections were mounted onto gelatin-coated glass slides and coverslipped with 50% (v/v) glycerol and 2.5% (w/v) triethylenediamine (anti-fading reagent) in PBS. Hybridization with the sense probe showed no signals stronger than background.

**Table 1:** **Antibodies used in the present study**.

Antigen	Host	Reference or Manufacturer		Concentration
**Primary antibodies**
GFP	Guinea pig	Nakamura et al. (2008)		1 μg/ml
VGluT1	Rabbit	Hioki et al. (2003)		1 μg/ml
VGluT2	Rabbit	Hioki et al. (2003)		1 μg/ml
VGAT	Rabbit	131003; Synaptic Systems, Göttingen, Germany		1 μg/ml
VGAT	Guinea pig	131004; Synaptic Systems		1 μg/ml
PV	Rabbit	PV-Rb-Af750; Frontier Institute, Hokkaido, Japan		1 μg/ml
SOM	Rabbit	T-4103; Peninsula Laboratories, Belmont, CA, USA		1 μg/ml
VIP	Rabbit	20077; ImmunoStar, Hudson, WI, USA		1:500
PSD95	Mouse	124011; Synaptic Systems		1 μg/ml
Gephyrin	Mouse	147021; Synaptic Systems		1:1000
NeuN	Mouse	MAB377; Merck Millipore, Darmstadt, Germany		10 μg/ml

**Antigen**	**Host**	**Fluorescence**	**Manufacturer**	**Concentration**

**Fluorescence-conjugated secondary antibodies**
Rabbit IgG	Goat	Alexa Fluor 488	A-11034; Life Technologies, Carlsbad, CA, USA	5 μg/ml
Rabbit IgG	Goat	Alexa Fluor 647	A-21245; Life Technologies	5 μg/ml
Guinea pig IgG	Goat	Alexa Fluor 488	A-11073; Life Technologies	5 μg/ml
Guinea pig IgG	Goat	Alexa Fluor 647	A-21450; Life Technologies	5 μg/ml
Mouse IgG	Goat	Alexa Fluor 568	A-11031; Life Technologies	5 μg/ml
Mouse IgG	Goat	Alexa Fluor 647	A-21236; Life Technologies	5 μg/ml

The fluorescence-labeled sections were observed under the Axiophot epifluorescence microscope (Carl Zeiss, Oberkochen, Germany) with appropriate filter sets. The numerical data were acquired from 18 vertical cortical strips (0.8–1.2 mm wide × cortical depth) of three mice. Images were taken with a QICAM FAST digital monochrome camera (QImaging, Surrey, BC, Canada).

### Immunofluorescence Labeling and Fluorescence Cytochrome Oxidase (CO) Staining

Mice were deeply anesthetized and transcardially perfused with PBS. Animals were further perfused with 4% (w/v) formaldehyde in 0.1 M Na_2_HPO_4_ (adjusted to pH 7.4 with NaOH) and 0.9% (w/v) picric acid. The brains were then removed and postfixed in the same fixative at room temperature for 3 h. Following cryoprotection, brain blocks were cut into 40-μm-thick coronal sections on a freezing microtome, based on preliminary experiments showing that antibody penetration was insufficient with brain sections thicker than 40 μm.

Antibodies used for immunohistochemistry are listed in Table 1. All of the following incubations were performed at room temperature and followed by 3 rinses with PBS containing 0.3% (v/v) Triton X-100 (PBS-X) for 10 min each. The brain sections were incubated overnight with a mixture of primary antibodies in PBS-X containing 0.12% *λ*-carrageenan and 1% normal donkey serum (PBS-XCD). The sections were then incubated overnight with a mixture of secondary antibodies in PBS-XCD.

We developed a new procedure for fluorescence visualization of cytochrome oxidase (CO) activity based on the deposition of BT. Mice were perfused with 4% (w/v) formaldehyde in 0.1 M PB followed by postfixation in the same fixative for 3 h. After cryoprotection, brain blocks were cut into 30-μm-thick tangential sections. The sections were washed with 0.1 M PB and then reacted with PB containing 45 mg/ml of β-D-glucose, 0.3 mg/ml of cytochrome C (Sigma-Aldrich, St Louis, MO, USA), and 0.13 mM of BT at 37°C for 3 h. The sections were then incubated with Alexa Fluor 568-conjugated streptavidin (S-11226; Life Technologies, Carlsbad, CA, USA). The sections were finally counterstained with 1 μg/ml of 4′,6-diamidino-2-phenylindole (DAPI; D-1306; Life Technologies) in PBS-X. The sections were mounted onto gelatin-coated glass slides and coverslipped with 50% (v/v) glycerol and 2.5% (w/v) triethylenediamine in PBS.

### Image Acquisition and Somatodendritic Reconstruction

Serial sections were observed under a TCS SP8 confocal laser scanning microscope (Leica Microsystems, Wetzlar, Germany) equipped with HyD detectors. For somatodendritic reconstruction, image stacks were captured using a 20× dry objective lens (HCX PL APO, numerical aperture [NA] = 0.50; Leica) with the pinhole at 1.0 Airy disk unit and zoom factor at 1. The somata and dendrites of VIP+ neurons were three-dimensionally reconstructed in 5–7 serial coronal section images with a NeuroLucida computer-assisted neuron tracing system (Microbrightfield Inc., Colchester, VT, USA). The reconstruction images were quantitatively analyzed by polar histogram (McMullen et al., 1984) and Sholl analysis (Sholl, 1953) with the NeuroLucida-attached software NeuroExplorer (Microbrightfield).

The triple-immunostained images for observation of input sites were acquired with a 63× oil-immersion objective lens (HCX PL APO, NA = 1.40; Leica) with the pinhole at 1.0 Airy disk unit. The three-dimensional- (3D) image stacks were captured with the zoom factor at 10 for dendritic domains and at 20 for somatic domains. Alexa Fluor 488, Alexa Fluor 568, and Alexa Fluor 647 were excited with 488, 552 and 638 nm laser beams and observed through 500–580, 590–650 and 660–850 nm emission prism windows, respectively.

Images were then deconvolved with Huygens Essential software (version 3.7; Scientific Volume Imaging, Hilversum, Netherlands) with the following parameters: microscopic type, confocal; back projected pinhole diameter, 238 nm; lens objective NA, 1.4; lens immersion refractive index, 1.515; medium refractive index, 1.515; excitation wavelength, 488, 552, or 638 nm; emission wavelength, 519, 603, or 668 nm; vertical mapping function, log; algorithm, classical maximum likelihood estimation; background estimation mode, lowest; relative background, 0%; maximum iteration number, 40; signal-to-noise ratio, 3; quality threshold, 0.1; iteration mode, fast; photobleaching correction, auto; and brick layout, auto. Morphological parameters (dendritic length, section area and somatic length) in the 3D-image stacks were measured by using software LSM 5 Image Examiner (Carl Zeiss).

Cortical layers in fluorescence-labeled sections were determined in reference to neuron-specific nuclear protein (NeuN) immunoreactivity and/or DAPI counterstaining.

### Synaptic Input Density Calculation

We selected 36 VIP+ neurons from 18 mice (6 neurons from 3 mice for each input analysis) according to the following criteria: (1) cell body was located in L2/3 of the S1BF but not near the border of L1 and L2; (2) the somatodendritic membrane was clearly visualized; (3) the infected neuron was well enough isolated for full reconstruction, and all dendritic branches derived from a single VIP+ neuron could be identified for manual tracing with NeuroLucida software. We then identified the longest branch from every primary dendrite based on the dendrogram, aided by NeuroExplorer software.

3D-image stacks of higher magnification were captured at the somatic membrane and dendritic membrane located 20 μm, and then every 50 μm, away from the somata. The somatic and dendritic surface area of interest was estimated as previously reported (Kameda et al., 2012; Hioki et al., 2013; also see Supplementary Figure 1). Briefly, the surface area of a dendritic segment could be calculated by the following formula:

(1) $$\begin{array}{l} Dendritic\;surface\;area[S_{d}]\;=\;\int_{0}^{l}\pi f(x)\text{d}x\;=\;\pi \int_{0}^{l}f(x)\text{d}x\;\\ \;=\;\pi \times Section\;area[A] \end{array}$$

The function *f*(*x*) is the dendritic diameter in the location *x*, and the value *l* is dendritic length. In the present study, we measured the sectional area [*A*] with LSM 5 Image Examiner software and calculated the dendritic surface area [*S_d_*] by multiplying π and [*A*]. To count the number of putative synaptic input sites on the somatodendritic membranes of VIP+ neurons, we observed the image stacks in 3D by using LSM 5 Image Examiner software. We then divided the number of putative synaptic inputs by the surface area of the somatodendritic membrane to obtain the density of synaptic inputs (/μm^2^).

Accounting for the possibility that the measurement of the cross-sectional area [*A*] was inaccurate, we also measured the dendritic surface area by using NeuroLucida and NeuroExplorer. After selecting 10 dendrites at each distance from the soma by using random number tables, we manually traced the dendrites with NeuroLucida and then calculated the dendritic surface area with NeuroExplorer. Because there was no significant difference between the two methods for estimating the dendritic surface area (Supplementary Table 1), we estimated all dendritic surface areas by multiplying π and [*A*] to obtain the number of inputs per surface area.

### Quantification of Axon Terminal Density

Immunofluorescence intensity in the S1BF for vesicular glutamate transporter 1 (VGluT1), VGluT2, or vesicular GABA transporter (VGAT) was quantified with ImageJ software (ver. 1.48; National Institutes of Health^1^) from the images of 6 cortical strips (151–212 μm wide × cortical depth) acquired under a confocal laser scanning microscope. Double-immunopositive puncta for VGAT and PV, SOM, or VIP in close contact with gephyrin immunoreactivity (putative axon terminals) were counted in the vertical direction every 30 μm from the pia mater to the white matter. The mean immunofluorescence intensities or axon terminal densities from the pia mater to the white matter were standardized to 1 arbitrary unit (AU) for each.

### Statistical Analysis

Multiple statistical comparisons were performed for Sholl analysis by the Bonferroni *post hoc* multiple comparison test following two-way analysis of variance (ANOVA), for comparisons of two groups by two-sided Student’s *t*-test, and for comparisons of three or more groups by Tukey’s multiple-comparison test after one-way ANOVA (Prism4.0c; GraphPad Software, San Diego, CA, USA).

## Results

### Somatodendritic Morphology of L2/3 VIP+ Neurons in the Mouse S1BF

To specifically visualize the somatodendritic membranes of VIP neurons, we injected AAV2/1-SynTetOff-FLEX-FGL into the S1BF of VIP-Cre knock-in mice (Figures 1A–A″,B). This Tet-Off gene expression system induced strong expression of FGL, a somatodendritic membrane-targeted GFP (Kameda et al., 2008, 2012), specifically in VIP+ neurons. The virus vector clearly visualized the morphology of the somatodendritic membranes as compared with VIP immunoreactivity (Figures 1C–E).

**Figure 2:**
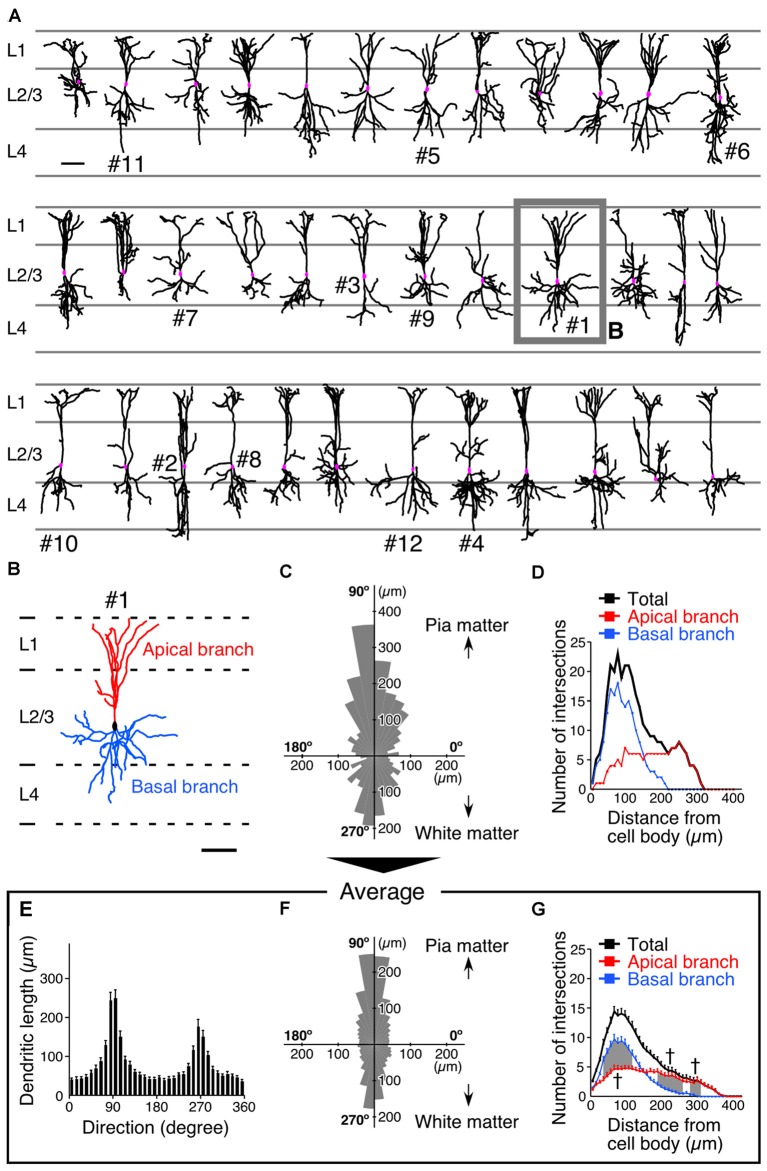
**Somatodendritic morphology of L2/3 VIP+ neurons in the S1BF. (A)** Somatodendritic morphology of VIP+ neurons in L2/3. GFP-labeled somatodendritic domains of VIP+ neurons were manually reconstructed with NeuroLucida, and all dendritic branches derived from a single VIP+ neuron were identified. Scale bar = 100 μm. **(B)** We defined primary dendrites with at least one branch extending in L1 as apical branches (red), and those oriented in other directions were defined as basal branches (blue). Scale bar = 100 μm. **(C)** Polar histogram of VIP+ neuron dendrites depicted in **(B)**. The total dendritic length in each 10° is summed as a pie-shaped wedge. Dendrites toward the pia mater are shown at 90°, while those toward the white matter are shown at 270°. **(D)** Sholl analysis of VIP+ neuron dendrites depicted in **(B)**. The number of dendrite intersections against the radial distance from the soma (every 10 μm) is plotted. Apical and basal branches are separately shown in red and blue, respectively. **(E,F)** Mean length of each radial bin from polar histograms of 36 VIP+ neurons shown in **(A)**. **(G)** Sholl analysis of 36 VIP+ neurons. Error bars, ± SEM. ^†^*p* < 0.05 using two-way ANOVA followed by Bonferroni *post hoc* multiple comparison test.

One week after the injection of the virus vector, we reconstructed the dendritic morphology of 36 VIP+ neurons and revealed that their dendrites exhibited bidirectional extension in the vertical orientation (Figures 2A,B). The length and orientation of dendrites of a representative reconstructed VIP+ neuron are shown in a polar histogram (Figure 2C). Two-thirds of the VIP+ neurons we reconstructed had more than two primary dendrites, defined as extending directly from the soma (two primary dendrites, 11; three, 16; four or more, 9). Even when VIP+ neurons had three or more primary dendrites, their polar histograms showed bidirectional extension in the vertical orientation (Figure 2C). VIP+ neurons located near the border of L1 and L2 were occasionally visualized, and these did not display bidirectional branch extension (Supplementary Figure 2). We excluded these neurons from the analysis of synaptic inputs. Although the number of VIP+ neurons is higher in the upper part of L2/3 than the lower one (Prönneke et al., 2015), the present sampling was slightly biased toward the lower part (Figure 2A). This may be because the virus injection site was slightly deeper than the center of L2/3, and we selected well-isolated neurons for full reconstruction according to the criteria as described in the “Materials and Methods” Section.

The averaged polar histogram from the 36 reconstructed VIP+ neurons indicated their bidirectional dendritic arborization (Figure 2F), and notably, the dendrites oriented toward the pia mater were significantly longer than those oriented toward the white matter (234.9 ± 21.6 μm vs. 150.6 ± 17.3 μm; mean ± SEM; *p* < 0.001 using one-way ANOVA followed by Tukey’s multiple-comparison test; Figures 2E,F). We defined primary dendrites ascending toward L1 as “apical branches”, with those oriented in other directions defined as “basal branches”. Quantification of their dendritic ramifications by Sholl analysis revealed markedly different bifurcation patterns between the apical and basal branches (Figure 2D). The number of the apical branches in the distal portion of VIP+ neuron dendrites was significantly larger than that of the basal branches (Figure 2G), reflecting abundant dendritic ramification in L1. On the other hand, in the proximal portion, the basal branches were more numerous than the apical ones (Figure 2G). These results suggest that their different ramifications are beneficial for the apical branches to receive abundant synaptic inputs in L1 as well as L2/3, and for the basal branches to largely receive inputs in L2–4 around the somata.

### Excitatory and Inhibitory Inputs to VIP+ Neurons along the Somatodendritic Axis

**Figure 3:**
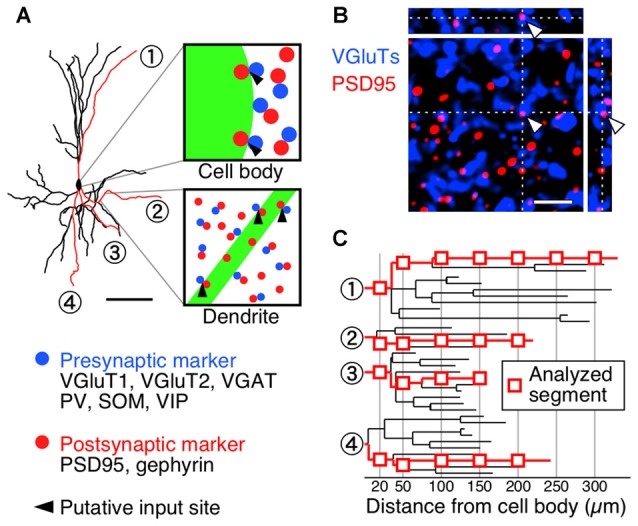
**Diagrams depicting procedure for counting synaptic inputs to VIP+ neurons. (A)** One week after virus injection, brain sections were triple-immunostained for GFP (green), presynaptic markers (blue) and postsynaptic markers (red). A presynaptic immunoreactive punctum (blue) was regarded as a putative synaptic input site only when the immunoreactivity for a postsynaptic marker (red) was observed at the somatodendritic side of the contact (black arrowheads). Scale bar = 100 μm.** (B)** Double-immunofluorescence image for vesicular glutamate transporters (VGluTs) (VGluT1 and VGluT2) and postsynaptic density protein 95 kDa (PSD95). To characterize the distribution of PSD95 in the mouse S1BF, we observed the immunoreactivities for VGluTs and PSD95 under a confocal laser scanning microscope. In the 3D-image stacks, most measured immunoreactivities formed close contacts (VGluTs/PSD95 = 96.1 ± 0.6% [2650/2757]; PSD95/VGluTs = 97.9 ± 0.4% [2650/2708]). Gephyrin was also previously reported as a reliable marker for inhibitory postsynaptic sites in the neocortex (Hioki et al., 2013) and in the trigeminal motor nucleus (Li et al., 2012). Arrowheads indicate input sites in *XY, XZ* and *YZ* planes. Scale bar = 2 μm. **(C)** High magnification images of the longest branches from primary dendrites were acquired under a confocal laser scanning microscope at the distances of 20 μm and every 50 μm away from the cell body (red rectangles).

We subsequently investigated the densities of inputs onto the somatodendritic membranes of each VIP+ neuron at subcellular spatial resolution. The brain sections were triple-immunostained for GFP and presynaptic and postsynaptic markers (Figure 3A). Immunoreactivities for VGluT1 and VGluT2 were used as corticocortical and thalamocortical excitatory presynaptic markers, respectively (Kaneko and Fujiyama, 2002; Fremeau et al., 2004), while VGAT immunoreactivity was used as an inhibitory presynaptic marker (Chaudhry et al., 1998). Since we previously reported that more than 95% of PV-, SOM- or VIP-immunoreactive puncta were positive for VGAT (Hioki et al., 2013), we used PV, SOM and VIP immunoreactivities to determine the GABAergic neuron subclass of each presynaptic structure. The postsynaptic structures for excitatory and inhibitory inputs were identified with immunoreactivities for postsynaptic density protein 95 kDa (PSD95) and gephyrin, respectively. Almost all PSD95-immunoreactive signals (96%) were closely contacted by VGluT1- or VGluT2-immunoreactive boutons and* vice versa* (98%), suggesting that PSD95 can be used as a useful marker for excitatory postsynaptic sites (Figure 3B). We also used gephyrin immunoreactivity as a marker for inhibitory postsynaptic sites, since it is localized at the postsynaptic side of inhibitory synapses (Li et al., 2012; Hioki et al., 2013). In the present study, the contact sites of presynaptic and postsynaptic immunoreactivities on the FGL-labeled membrane were considered putative synaptic input sites (Figure 3A).

**Figure 4:**
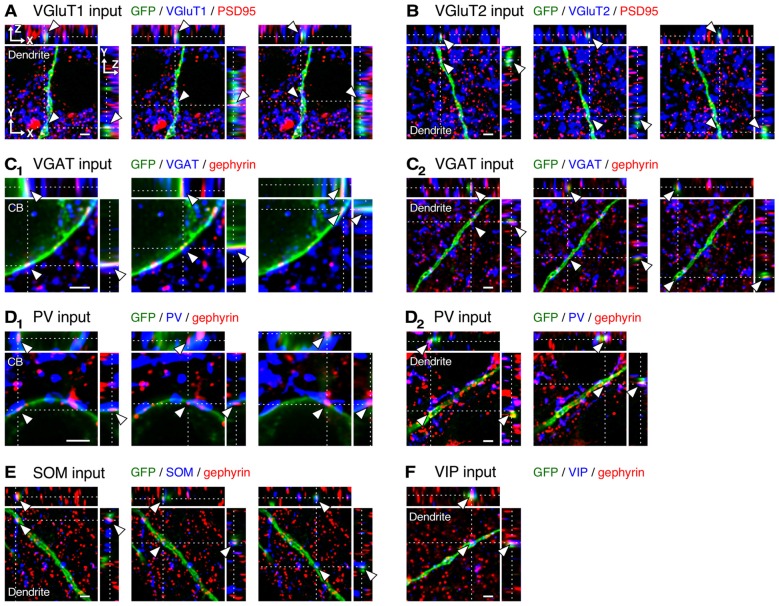
**Three-dimensional observation of VGluT1, VGluT2, vesicular GABA transporter (VGAT), parvalbumin (PV), somatostatin (SOM) and VIP inputs to L2/3 VIP+ neurons. (A–C_2_)** Orthogonal views of VGluT1 (**A**, distance of 200 μm from cell body, CB), VGluT2 (**B**, distance of 250 μm from CB) and VGAT inputs (**C_2_**, distance of 200 μm from CB) on the dendritic membrane of VIP+ neurons, and those of VGAT inputs on the somatic membrane **(C_1_**) are shown. **(D_1_–F)** Orthogonal views of PV (**D_2_**, distance of 20 μm from CB), SOM (**E**, distance of 100 μm from CB) and VIP inputs (**F**, distance of 200 μm from CB) on the dendritic membrane of VIP+ neurons, and those of PV inputs on the somatic membrane **(D_1_**) are shown. Arrowheads indicate the putative synaptic input sites on VIP+ neuron membrane. Scale bars = 2 μm.

For the analysis of the dendritic segment, we identified the longest branch from every primary dendrite based on the dendrogram (Figure 3C) and quantified appositions of presynaptic structures to the dendritic branch at 20 μm and every 50 μm away from the soma (Figure 3C). The 3D image stacks of triple-immunofluorescence stained sections were obtained under a confocal laser scanning microscope, and the putative synaptic input sites on the somata and dendrites were counted (Figure 4).

**Figure 5:**
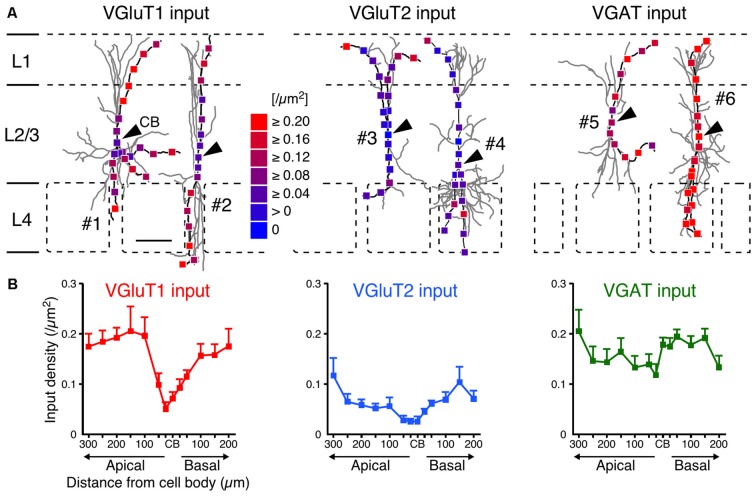
**VGluT1, VGluT2 and VGAT input densities to L2/3 VIP+ neurons. (A)** Examples of VGluT1 (left), VGluT2 (middle) and VGAT inputs (right). Arrowheads indicate CB #1–6 correspond to #1–6 in Figure 2A. Scale bar = 100 μm. **(B)** VGluT1, VGluT2 and VGAT input densities are plotted as a function of distance along the apical and basal branches. To calculate the input densities to VIP+ neurons, we counted the number of input sites and divided them by the surface areas. VGluT1 inputs were more frequently observed in the distal dendrites than in the somata (left). VGluT2 inputs (middle) also increased from the somata to the distal dendrites, but VGluT2 input density was less than half of VGluT1 input density. VGAT input density, on the other hand, did not significantly differ among distances from the somata (right). Input densities were not significantly different between the apical and basal branches at equivalent distances from the somata. Error bars, ± SEM.

**Figure 6:**
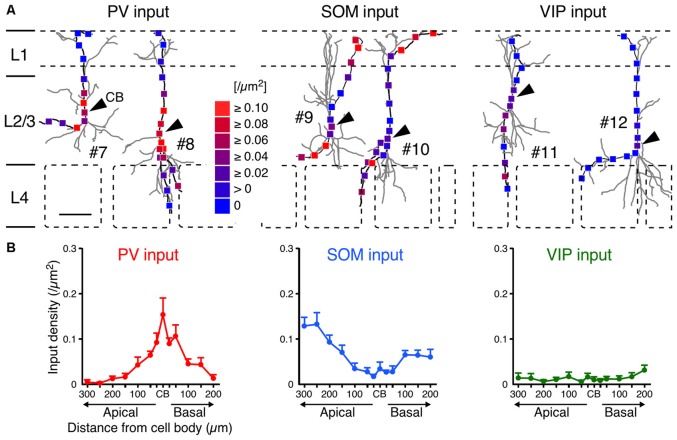
**PV, SOM and VIP input densities to L2/3 VIP+ neurons. (A)** Examples of PV (left), SOM (middle) and VIP inputs (right). Arrowheads indicate CB. #7–12 correspond to #7–12 in Figure 2A. Scale bar = 100 μm. **(B)** PV, SOM and VIP input densities are plotted as a function of distance along the apical and basal branches. PV inputs (left) were more frequently observed in the somata and proximal dendrites than in distal dendrites, whereas SOM inputs (middle) showed the opposite pattern. VIP inputs (right) were few and showed no significant differences among subcellular input domains. Input densities were not significantly different between the apical and basal branches at equivalent distances from the somata. Error bars, ± SEM.

After observing and counting the synaptic contact sites (Figure 4; Supplementary Table 2), we calculated the input densities per surface area (Figures 5, 6) and per dendritic length (Supplementary Table 2) for the presynaptic markers at each sampling point. Corticocortical excitatory inputs (VGluT1 inputs) were more densely distributed on the distal dendrites than on the somata (Figure 5). Thalamocortical excitatory input (VGluT2 input) density also increased from the somata to the distal dendrites, but VGluT2 input density was 25%–67% of VGluT1 input density at equivalent distances from the somata (Figure 5). These observations show that VIP+ neurons receive both excitatory inputs mainly in the distal dendrites, and their somata are much less innervated by excitatory neurons. Inhibitory input (VGAT input) density, on the other hand, did not significantly differ along the somatodendritic axis (Figure 5).

Although the somatodendritic membrane of VIP+ neurons was evenly covered by VGAT inputs regardless of the distance from the somata, unequal distribution patterns were found when we focused on inputs from each subgroup of GABAergic inhibitory neurons: PV+, SOM+ and VIP+ neurons. Inputs from PV+ neurons (PV inputs) were more densely distributed on the somata and proximal dendrites than on the distal dendrites, whereas inputs from SOM+ neurons (SOM inputs) showed the opposite pattern (Figure 6). Notably, inputs from VIP+ neurons (VIP inputs) were rarely found and the few that were present showed no significant difference along the somatodendritic axis (Figure 6), indicating that L2/3 VIP+ neurons in the S1BF have few reciprocal connections.

### Perisomatic or Distal-Dendritic Targeting of Synaptic Inputs to VIP+ Neurons

**Figure 7:**
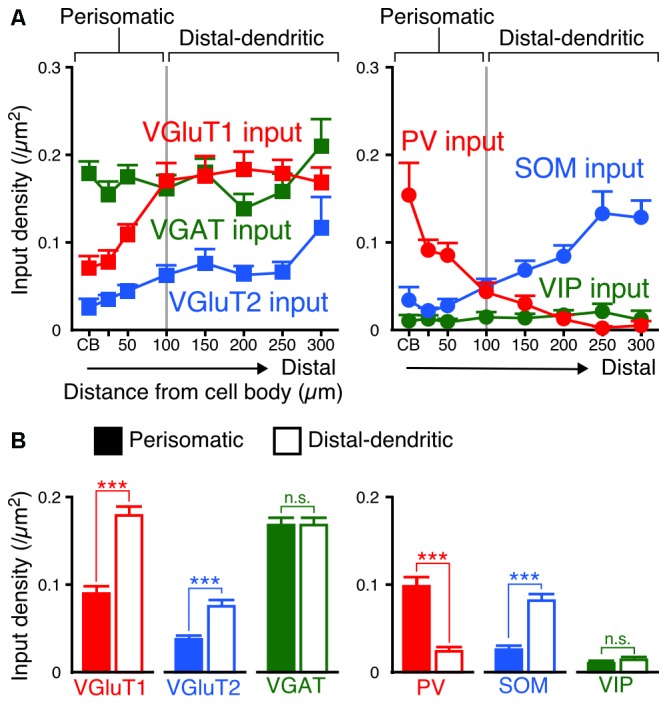
**Input densities on the perisomatic and distal-dendritic compartments of VIP+ neurons. (A)** The input densities to the apical and basal branches were pooled. Both VGluT1 and VGluT2 input densities were higher in the distal dendrites than in the somata and proximal dendrites (VGluT1 input, *p* < 0.0001; VGluT2 input, *p* = 0.0024 using one-way ANOVA). VGAT input density did not show significant differences among distances from the somata (*p* = 0.3231 using one-way ANOVA). PV inputs were more densely observed in VIP+ neuron somata than in their distal dendrites, whereas SOM inputs showed the opposite pattern (PV input, *p* < 0.0001; SOM input, *p* < 0.0001 using one-way ANOVA). The mutual connections among VIP+ neurons were few and VIP input density did not significantly differ among distances from the somata (*p* = 0.8961 using one-way ANOVA). The somatodendritic domain of VIP+ neurons could be divided by these innervation patterns at a distance of 100 μm from the somata into the “perisomatic” and “distal-dendritic” compartments. **(B)** Comparison of input densities to the perisomatic and distal-dendritic compartments. VGluT1 input, *p* < 0.0001; VGluT2 input, *p* < 0.0001; VGAT input, *p* = 0.9079; PV input, *p* < 0.0001; SOM input, *p* < 0.0001; VIP input, *p* = 0.3018 using two-tailed Student’s *t*-test. Six neurons from three mice for each input. Error bars, ± SEM. ***p < 0.001; n.s., not significant.

Since the densities of inputs to the apical and basal branches of VIP+ neurons were similar with no significant differences at equivalent distances from the somata (Figures 5, 6), we combined the data of input densities obtained from the apical and basal branches (Figure 7A). VGluT1 inputs were 1.4–2.9-fold denser than VGluT2 inputs, and both excitatory input densities in the distal dendrites were higher than those in the somata (Figure 7A). In particular, VGluT1 input density increased linearly from the somata to the 100-μm position on the dendrites; however, it did not obviously increase in the farther distal dendritic segment (0.168–0.183/μm^2^ in the segment of 100–300 μm from the somata; Figure 7A). Although VGAT input density did not significantly differ along the somatodendritic axis, more PV inputs were distributed in the somatodendritic segment within 100 μm from the somata than in the farther distal segment (Figure 7A). On the other hand, SOM inputs were predominantly observed at the dendritic segment distal to the 100-μm position (Figure 7A). The density of PV inputs to the somata was comparable to that of SOM inputs to the distal dendrites.

We divided the somatodendritic axis of VIP+ neurons into two parts at the 100-μm position where the population of presynaptic cell types gradually changed: the “perisomatic” (<100 μm from the soma) and “distal-dendritic” compartments (≥100 μm from the soma). Comparisons of averaged input densities between the compartments (Figure 7B) showed that VGluT1, VGluT2 and SOM input densities were significantly higher in the distal-dendritic compartment, whereas PV input density was higher in the perisomatic compartment. The proportions of PV, SOM and VIP inputs of total VGAT inputs to the perisomatic compartment were 57.9%, 18.3% and 6.6%, whereas those to the distal-dendritic compartment were 16.7%, 46.7% and 8.8%, respectively. This result indicates that the perisomatic and distal-dendritic compartments of L2/3 VIP+ neurons in the S1BF receive distinct populations of excitatory and inhibitory inputs.

### Input Density Correction with Axon Terminal Density in the Vertical Direction

**Figure 8:**
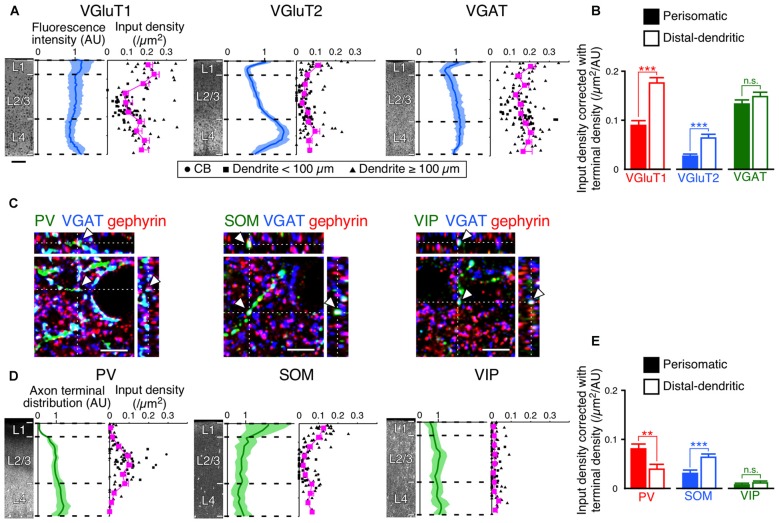
**Input densities corrected with total densities of axon terminals. (A)** VGluT1-, VGluT2- and VGAT-immunofluorescence intensities across layers are shown in blue (mean ± SD), where each mean intensity value across layers from the pia mater to the white matter was standardized as 1 arbitrary unit (AU). Scatter diagrams show input densities and cortical depths (black). The cortical depth from L1 to L4 is divided into 10 bins, and the mean input densities are plotted (magenta). Scale bar = 100 μm (also applied to **D**). **(B)** VGluT1, VGluT2 and VGAT input densities corrected with axon terminal intensities. The input densities were divided by the axon terminal intensities at equivalent cortical depths. VGluT1 input, *p* < 0.0001; VGluT2 input, *p* < 0.0001; VGAT input, *p* = 0.1485 using two-tailed Student’s *t*-test. Six neurons from three mice for each input. **(C)** PV-, SOM-, and VIP-positive axon terminals. PV-, SOM- or VIP-immunoreactive puncta were regarded as axon terminals only when the puncta were also positive for VGAT. The double-positive puncta apposing gephyrin-immunoreactive dots were counted to determine axon terminal densities (arrowheads). Scale bars = 5 μm. **(D)** PV-, SOM- and VIP-positive axon terminal densities across layers are shown in green (mean ± SD), where each mean density value across layers from the pia mater to the white matter was standardized as 1 AU. Scatter diagrams show input densities and cortical depths (black). The cortical depth from L1 to L4 is divided into 10 bins, and the mean input densities are plotted (magenta). **(E)** PV, SOM and VIP input densities corrected with axon terminal densities. The input densities were divided by the axon terminal densities at equivalent cortical depths. PV input, *p* = 0.001; SOM input, *p* < 0.0001; VIP input, *p* = 0.2155 using two-tailed Student’s *t*-test. Error bars, ± SEM. **p < 0.01; ***p < 0.001; n.s., not significant.

The innervation patterns to VIP+ neurons varied between the perisomatic and distal-dendritic compartments. We hypothesized that the unequal distribution patterns resulted from the difference of axon terminal densities in different layers. For example, since thalamocortical neurons project more abundantly to L4 and L1 than to L2/3, the dense VGluT2 inputs in the distal-dendritic compartment, frequently arborized in L1 and L4, could be ascribed to this uneven thalamocortical axonal arborization. To address this issue, we compared the scatter diagrams of input densities along the vertical somatodendritic axis of L2/3 VIP+ neurons with the densities of VGluT1-, VGluT2-, VGAT-, PV-, SOM- and VIP-positive axon terminals (Figures 8A,D). Because VGluT1, VGluT2 and VGAT immunoreactivities were exclusively localized in presynaptic structures in axons, we obtained total densities of VGluT1-, VGluT2- and VGAT-immunoreactive axon terminals vertically through L1–6 in the S1BF from their immunofluorescence intensities (Figure 8A). On the other hand, PV, SOM and VIP immunostaining labeled somata and dendrites as well as axons. Therefore, we counted puncta co-labeled with VGAT immunoreactivity and apposed to gephyrin immunoreactive puncta as PV+, SOM+ or VIP+ axon terminals through L1–6 to obtain their total axon terminal densities (Figures 8C,D).

If presynaptic boutons randomly form synapses on the somatodendritic membrane of VIP+ neurons, the synaptic input densities should show vertical distributions proportional to the total axonal bouton densities. However, despite the fact that VGluT1 immunofluorescence intensity in L4 was slightly less than that in L2/3, VGluT1 inputs to VIP+ neurons were denser in L4 than in L2/3; the axon terminal density of PV+ neurons almost monotonically increased from L1 to L4, but PV input density in L4 was less than that in L2/3 (Figures 8A,D). VGluT1 and PV input densities to VIP+ neurons were obviously disproportionate to their total axon terminal densities.

We then divided the input densities by the total axon terminal densities at equivalent cortical depths (Figures 8B,E). If the distribution of the input densities simply reflects that of the axon terminal densities, the corrected densities should be comparable between the somatodendritic compartments. Not only the corrected densities of VGluT1 and PV inputs, but also those of VGluT2 and SOM inputs were significantly different between the compartments (Figures 8B,E). These results indicate that the observed non-uniform patterns of the inputs to VIP+ neurons result from preferential innervation to the distinct subcellular compartments of VIP+ neurons.

### Inputs to the Distal-Dendritic Compartment in Barrels/Septa of L4

**Figure 9:**
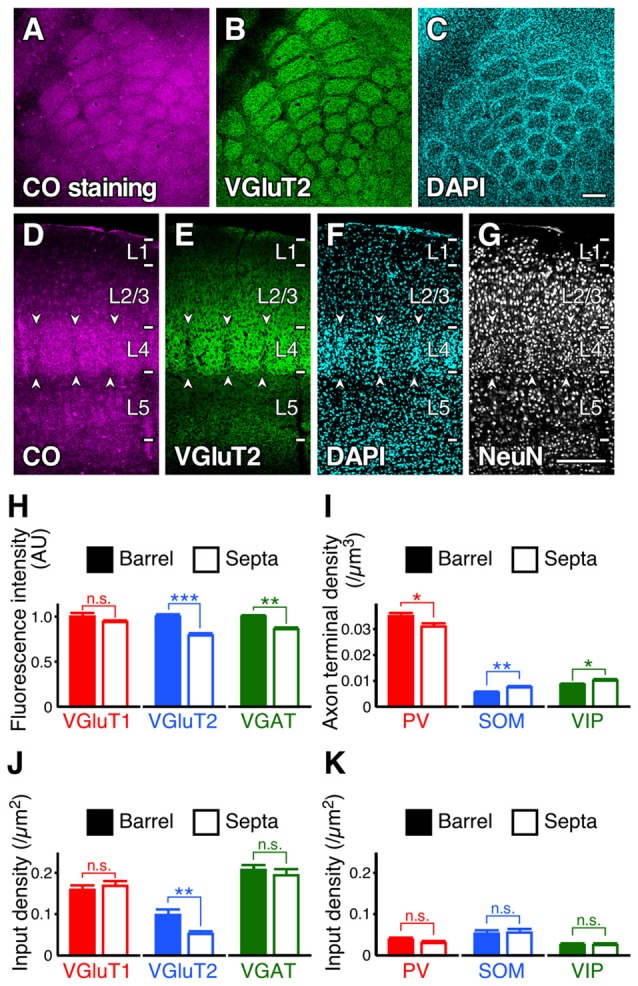
**Input densities to L2/3 VIP+ neuron dendrites in barrels and septa of L4. (A–G)** Tangential and coronal sections with CO-fluorescence staining **(A,D)**, immunofluorescence staining for VGluT2 **(B, E)** and NeuN **(G)**, and DAPI counterstaining **(C,F)**. Barrels and septa could be identified through comparison with VGluT2 immunofluorescence and DAPI counterstaining. Arrowheads indicate septa. Scale bars = 200 μm. **(H,I)** Immunofluorescence intensities **(H)** and axon terminal densities **(I)** in barrels and septa. Mean immunofluorescence intensities in barrels were standardized as 1 AU **(H)**. VGluT1, *p* = 0.244; VGluT2, *p* < 0.0001; VGAT, *p* = 0.002; PV, *p* = 0.0361; SOM, *p* = 0.0097; VIP, *p* = 0.0495; nine observed regions of the centers of barrels or septa for each axon terminal from three mice using two-tailed Student’s *t*-test. **(J,K)** Input densities onto the dendrites in barrels and septa (~30 dendrites from three mice for each input). VGluT1 input, *p* = 0.5388; VGluT2, *p* = 0.0026; VGAT, *p* = 0.5047; PV, *p* = 0.2814; SOM, *p* = 0.8237; VIP, *p* = 0.7617 from three mice using two-tailed Student’s *t*-test. Error bars, ± SEM. *p < 0.05; **p < 0.01; ***p < 0.001; n.s., not significant.

Subsequently, we evaluated the densities of inputs to VIP+ neuron dendrites in the tangential plane. In L4 of the rodent S1BF, barrels and inter-barrel septa can be histochemically identified by endogenous CO activity (Land and Simons, 1985). We developed a new procedure for fluorescence visualization of CO activity to identify barrels and septa through comparison with VGluT2 immunofluorescence and DAPI counterstaining (Figures 9A–G). VGluT2-immunoreactivity, which is more densely distributed in barrels than in septa (Figures 9B,E; Liguz-Lecznar and Skangiel-Kramska, 2007), as well as VGAT- and PV-positive axon terminals, showed significantly higher densities in barrels than in septa (Figures 9H,I). In contrast, SOM- and VIP-positive axon terminals were more abundant in septa (Figure 9I). Although we extended the columnar compartments to L2/3 and compared the fluorescence intensities for VGluT1, VGluT2, and VGAT, there was no significant difference between the extended barrels and septa in L2/3 (Table 2).

**Table 2:** **Immunofluorescence intensities for vesicular glutamate transporter 1 (VGluT1), VGluT2 and vesicular GABA transporter (VGAT) in L2/3 and L4 of the mouse primary somatosensory cortex barrel field (S1BF)**.

	Barrel column	Septal column	*p* value
	**VGluT1**		
	
Upper L2/3	1.00 ± 0.04	1.04 ± 0.03	0.468
Middle L2/3	1.00 ± 0.04	1.05 ± 0.03	0.303
Lower L2/3	1.00 ± 0.03	1.03 ± 0.03	0.553
L4	1.00 ± 0.04	0.94 ± 0.02	0.244

	**VGluT2**		
	
Upper L2/3	1.00 ± 0.05	1.00 ± 0.05	0.962
Middle L2/3	1.00 ± 0.04	1.00 ± 0.04	0.973
Lower L2/3	1.00 ± 0.03	1.02 ± 0.03	0.623
L4	1.00 ± 0.01	0.79 ± 0.02	<0.0001

	**VGAT**		
	
Upper L2/3	1.00 ± 0.02	0.91 ± 0.10	0.382
Middle L2/3	1.00 ± 0.03	0.99 ± 0.03	0.813
Lower L2/3	1.00 ± 0.03	0.92 ± 0.10	0.451
L4	1.00 ± 0.02	0.86 ± 0.03	0.002

*Mean fluorescence intensities ± SEM in the barrel and septal columns are shown. We extended the barrel and septal columns to L2/3, and measured the immunofluorescence intensities for VGluT1, VGluT2 and VGAT in the upper, middle and lower parts of L2/3 by dividing into 3 bins. Mean immunofluorescence intensities in the barrel column were standardized as 1 AU. Whereas immunofluorescence intensities for VGluT2 and VGAT were higher in barrel than in septa of L4, those in the barrel and septal columns of L2/3 were not significantly different*.

Because the axon terminal densities were different between barrels and septa (except for VGluT1) in L4, we compared the input densities to the dendrites of L2/3 VIP+ neurons at the centers of barrels and septa. L2/3 VIP+ neuron dendrites in the middle part of L4 are assumed to be the distal-dendritic compartment (≥100 μm) because the vertical thickness of L4 in the S1BF is around 200 μm. VGluT2 inputs were more frequently observed in barrels than in septa (Figure 9J). Although VGluT2 inputs were not proportional to the vertical distribution of thalamocortical axon terminals (Figures 8A,B), these inputs were to some extent influenced by the tangential distribution of VGluT2-positive axon terminals. However, the densities of VGAT, PV, SOM and VIP inputs to VIP+ neurons did not differ significantly between barrels and septa (Figures 9J,K). In addition, while the density of PV+ axon terminals was higher than that of SOM+ terminals in L4 of the S1BF (Figure 9I), PV input density was less than that of SOM inputs (PV inputs, 0.034 ± 0.004 /μm^2^; SOM inputs, 0.054 ± 0.006 /μm^2^; mean ± SEM; *n* = 61 and 60, respectively; *p* = 0.0039 using two-tailed Student’s *t*-test). This is likely because the distal-dendritic compartment preferentially received SOM inputs, but not PV inputs. Thus, these results suggest that inhibitory input densities to the distal-dendritic compartment of VIP+ neurons are largely independent of the axon terminal densities around the somatodendritic domains.

## Discussion

**Figure 10:**
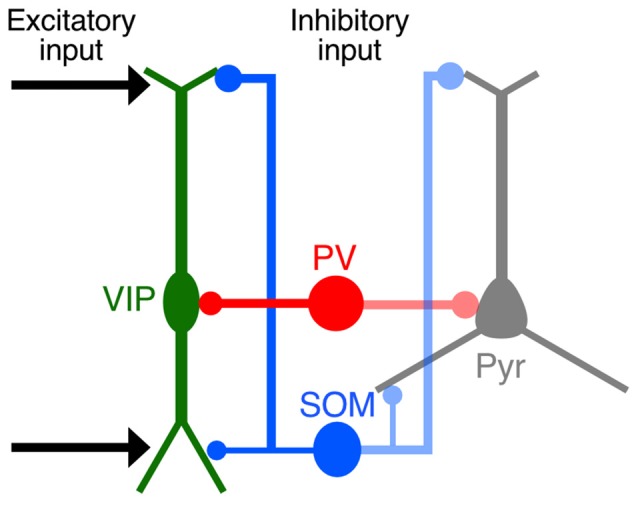
**Schematic diagram summarizing synaptic inputs to VIP+ neurons.** Both cortical and thalamic excitatory neurons innervate the distal-dendritic compartment of VIP+ neurons, and the distal-dendritic compartment is inhibited mainly by SOM+ neurons. The perisomatic compartment of VIP+ neurons is predominantly innervated by PV+ neurons. These input patterns to the subcellular compartments of VIP+ neurons resemble those of pyramidal cells.

Our quantitative imaging analyses focused on the distribution of synaptic inputs to L2/3 VIP+ neurons in the S1BF at subcellular resolution and revealed that synaptic inputs to VIP+ neurons had preferences for certain somatodendritic compartments, similar to the pattern of innervation to pyramidal cells (Figure 10). The distal-dendritic compartment of VIP+ neurons received more excitatory inputs and SOM inputs, whereas the perisomatic compartment was predominantly innervated by PV+ neurons. Preferential targeting to the different compartments of VIP+ neurons by SOM+ and PV+ neurons would provide different inhibitory effects on the regulation of VIP+ neuron activity. The present findings show a precise configuration of site-specific inputs to VIP+ neurons.

### Technical Considerations

In the present study, we investigated the synaptic inputs onto the perisomatic and distal-dendritic compartments of VIP+ neurons by confocal laser scanning microscopy with the aid of postsynaptic markers such as PSD95 and gephyrin. However, elucidation of the absolute number of synapses on VIP+ neurons would require an electron microscopy study, because the present light microscopy method may lead to false-positive and/or -negative errors. Although previous electron microscopy studies have revealed that VIP+ neurons receive synaptic inputs from pyramidal cells (Staiger et al., 2002), thalamic neurons (Staiger et al., 1996), and PV+ neocortical GABAergic neurons (Staiger et al., 1997), questions remain about the positional preferences of excitatory and inhibitory inputs to VIP+ neurons. Therefore, systematic analysis of synaptic inputs to VIP+ neurons by electron microscopy is expected to provide verification of the present findings. In addition, a further technical consideration for future studies is whether PV+, SOM+ or VIP+ boutons sufficiently label the presynaptic structures of each type of GABAergic neuron.

One electron microscopy study of the hippocampus determined the numbers of excitatory and inhibitory inputs to GABAergic neurons expressing calretinin (CR), which may correspond to VIP+ neurons (Gulyás et al., 1999). Serial electron microscopy in the rat hippocampus revealed that only 20% of synaptic inputs to CR+ neurons were GABAergic (Gulyás et al., 1999), inconsistent with the present finding. Gulyás et al. (1999) also reported that the number of excitatory synapses on CR+ neuron somata was 1.4-times greater than that of inhibitory synapses. However, in the visual cortex, an electron microscopy study demonstrated that synapses on VIP+ neuron somata were mostly symmetric (Hajós and Zilles, 1988), suggestive of inhibitory inputs. Furthermore, PV+ boutons extensively formed symmetric synapses on VIP+ neuron somata in the S1 (Staiger et al., 1997). These previous reports suggest that VIP+ neuron somata in the neocortex might receive more inputs from inhibitory neurons than do CR+ neuron somata in the hippocampus.

### Dendrite-Targeting Excitatory Inputs to L2/3 VIP+ Neurons

Both corticocortical and thalamocortical excitatory neurons exhibited stronger innervation of the distal-dendritic compartment of VIP+ neurons. In light of abundant dendritic ramification in L1, the present findings suggest that L2/3 VIP+ neurons can be strongly recruited by the excitatory inputs distributed in L1. L1 excitatory inputs can thereby elicit “feedforward disinhibition” on pyramidal cells by reducing the activities of SOM+ and PV+ neurons through VIP+ neuron activation (Pfeffer, 2014).

The origin of corticocortical excitatory inputs to the S1BF stems not only from pyramidal cells within the S1 but also from long-projecting pyramidal cells, such as those in the secondary somatosensory (S2) and primary motor (M1) cortices (White and DeAmicis, 1977; Fabri and Burton, 1991; Veinante and Deschênes, 2003; Kinnischtzke et al., 2014). For instance, VIP+ neurons in the S1BF are strongly recruited by excitatory inputs from the M1, and their firing rates are increased during free whisking (Lee et al., 2013).

The S1BF receives thalamocortical projections from the ventral posteromedial thalamic nucleus (VPM). Electron microscopy showed that VIP+ neurons in L3 and L4 of the S1BF receive synaptic inputs from the VPM (Staiger et al., 1996). The S1BF also receives inputs from the rostral sector of the posterior thalamic nuclei (POm; Wimmer et al., 2010). The POm can be divided into anterior and posterior parts according to calbindin immunoreactivity. Anterior and posterior POm neurons send axons to L5 of the S1 and to L1 with wide arborization, respectively (Ohno et al., 2012). Furthermore, the motor thalamic nuclei provide axon fibers to the S1. The caudolateral portion of the ventral anterior-ventral lateral motor thalamic nuclei (VA/VL) sends axons to the middle layer of the S1, whereas the rostromedial portion of the VA/VL and the ventral medial nucleus (VM) have widespread projections to L1 of the S1 (Kuramoto et al., 2009, 2015). It is therefore assumed that the apical and basal branches of L2/3 VIP+ neurons receive synaptic inputs from different thalamic nuclei. Further study is necessary for elucidation of the thalamic nuclei that affect VIP+ neuronal activity.

### SOM+ Neurons Innervate the Distal-Dendritic Compartment of L2/3 VIP+ Neurons

In addition to excitatory inputs, SOM+ inhibitory inputs were also frequently found in the distal-dendritic compartment of VIP+ neurons. Most SOM+ neurons correspond to L1-targeting Martinotti cells (Kawaguchi and Kubota, 1996; Wang et al., 2004; Ma et al., 2006; McGarry et al., 2010) and are strongly innervated by VIP+ neurons (Dalezios et al., 2002; Staiger et al., 2004; Lee et al., 2013; Pfeffer et al., 2013; Pi et al., 2013; Fu et al., 2014; Zhang et al., 2014). Thus, VIP+ and SOM+ neurons have strong reciprocal connections.

Local dendritic inhibition of pyramidal cells block dendritic calcium spikes (Larkum et al., 1999; Murayama et al., 2009) and gates dendritic electrogenesis driving burst spiking in pyramidal cells (Lovett-Barron et al., 2012). Thus, our present observations support the possibility that SOM+ neurons shunt local activation evoked by excitatory inputs in VIP+ neuron dendrites.

There remains an unidentified population of inhibitory inputs (28% of VGAT inputs) that contacts the distal-dendritic compartment. Even though our light microscopic observation could over- or under-estimate input numbers, a substantial number of these unidentified GABAergic inputs likely contribute to dendritic inhibition of VIP+ neurons together with SOM inputs. GABAergic neurons that do not have PV, SOM, or VIP, such as neurogliaform cells and L1 interneurons (Rudy et al., 2011; Jiang et al., 2013; Lee et al., 2015), are thought to inhibit the distal-dendritic compartment of VIP+ neurons, though the chemical characteristics of cells inhibiting VIP+ neuron dendrites could not be entirely determined in the present study.

### Perisomatic Inputs to L2/3 VIP+ Neurons from PV+ Neurons

While the perisomatic compartment of L2/3 VIP+ neurons in the S1BF received fewer excitatory inputs, it did receive abundant PV inputs. PV+ perisomatic boutons on VIP+ neurons form symmetric synapses as shown by electron microscopy (Staiger et al., 1997). Because VIP+ neurons also innervate the somatic compartment, not the dendritic compartment, of PV+ neurons (Dávid et al., 2007; Hioki et al., 2013), these two subtypes are reciprocally connected with each other’s perisomatic compartments. Since perisomatic inhibition strongly suppresses the generation of action potentials in target cells (Miles et al., 1996; Freund and Katona, 2007; Strüber et al., 2015), VIP+ and PV+ neurons can effectively and rapidly inhibit each other’s firing with regard to electrical distance through axosomatic connections.

In addition, although L2/3 VIP+ neurons spread their dendrites almost vertically from L1 to L4, they received PV inputs mainly in L2/3 (Figure 8D). On the other hand, PV+ neurons spread their axons horizontally or concentrically (Somogyi et al., 1983; Kawaguchi and Kubota, 1997). Therefore, PV inputs to VIP+ neurons likely originate mostly from neighboring L2/3 PV+ neurons. L2/3 PV+ neurons receive sensory-evoked feedforward excitation from L4 and are recruited more rapidly than SOM+ neurons in the primary auditory cortex (Li et al., 2014). This fast response by PV+ neurons provides feedforward inhibition to pyramidal cells and rapidly refines auditory representation. Thus, PV inputs to the perisomatic compartment of VIP+ neurons might also contribute to the enhancement of the receptive field contrast of pyramidal cells in the neocortex.

### Heterogeneity of Chemically-Classified Subgroups of GABAergic Neurons

VIP+ neurons include subpopulations that express CR, cholecystokinin (CCK), choline acetyltransferase, or corticotropin-releasing factor (Eckenstein and Baughman, 1984; Bayraktar et al., 1997; Xu et al., 2010; Kubota et al., 2011). VIP+/CR− and VIP+/CR+ neurons exhibit different firing properties (Porter et al., 1998), and a small subpopulation of VIP+ neurons expressing CCK shows small basket cell-type morphology (Kawaguchi and Kondo, 2002; Markram et al., 2004). Recently, it was demonstrated that VIP+ neurons could be categorized by the layer in which they are located because the morphological and electrophysiological features of VIP+ neurons in L2/3 are significantly different from those in deeper layers (Prönneke et al., 2015). Our data showed significant differences in input densities between the perisomatic and distal-dendritic compartments of L2/3 VIP+ neurons. Future study is necessary to determine whether different VIP+ neuronal subpopulations and/or VIP+ neurons in deeper layers display different innervation patterns.

PV+ and SOM+ neurons also display diversity, especially in morphology and chemical characteristics. PV+ neurons mostly correspond to fast-spiking basket cells, though axo-axonic chandelier cells and multipolar bursting cells, two minor populations, also express PV (Kawaguchi and Kondo, 2002; Blatow et al., 2003; Markram et al., 2004; Burkhalter, 2008). About half of SOM+ neurons express preprodynorphin, one of the precursors of opioid peptides, and other smaller subpopulations express CR, neuropeptide Y, and/or nitric oxide synthase (Xu et al., 2006, 2010; Sohn et al., 2014). While most SOM+ neurons are morphologically characterized as L1-targeting Maltinotti cells, the axons of those in L4 do not target L1 but rather remain within L4 (Ma et al., 2006; Xu et al., 2013). Although the present analysis could not distinguish the presynaptic structures of these subpopulations of PV+ and SOM+ neurons, the vast majority of PV and SOM inputs to VIP+ neurons are assumed to be derived from fast-spiking basket cells and Maltinotti cells, respectively.

### Structural Basis for Input-Output Transformation in VIP+ Neurons

The neuron types that innervate VIP+ neurons have been explored by electrophysiological approaches (Porter et al., 1998; Rozov et al., 2001; Lee et al., 2013; Pfeffer et al., 2013; Zhang et al., 2014; Ji et al., 2016), c-Fos induction (Bubser et al., 1998; Lecrux et al., 2011), and retrograde labeling of monosynaptic inputs with rabies virus (Fu et al., 2014; Zhang et al., 2014). Although these approaches have determined cell-to-cell connectivity and connection strength, limitations on the spatial resolution in these previous studies have hindered the elucidation of the subcellular localization of synaptic inputs to VIP+ neurons. Some have proposed that the postsynaptic potential at the soma is not just the sum of the potentials in dendrites generated by synaptic inputs, but the result of adjacent synapses on dendrites locally interacting with each other (Koch et al., 1983; Koch and Segev, 2000). In addition, the proximal and distal dendritic portions of bipolar cells exhibit different properties in action potential backpropagation and calcium dynamics due to the presence of I_A_-type potassium currents (Goldberg et al., 2003). Interestingly, the boundary of these different postsynaptic properties in bipolar cell dendrites (100 μm away from the somata) coincides with the transition zone of the input patterns demonstrated in the present study. These data suggest that the cell type-specific inputs to VIP+ neurons that preferred either the perisomatic or distal-dendritic compartment are integrated through discrete postsynaptic computation mechanisms.

## Author Contributions

JS, TK and HH designed the research; JS performed the research; JS, SO, NK, KN and HH contributed to development of the new AAV vector; JS analyzed the data; JS, KN and HH wrote the article; JS, SO, NK, KN and HH revised the manuscript.

## Funding

This work was supported by Grants-in-Aid from The Ministry of Education, Culture, Sports, Science and Technology (MEXT) and the Japan Society for the Promotion of Science (JSPS); for JSPS Fellows (13J01992 to JS); for Research Activity Start-up (16H07425 to JS); for Scientific Research (24790233 to NK, 25250006 to TK, 26713009 and 16H05128 to KN, and 16H04663 to HH); for Exploratory Research (15K14333 to HH); and for Scientific Research on Innovative Areas, “Mesoscopic Neurocircuitry” (23115101 to TK), “The Evolutionary Origin and Neural Basis of the Empathetic Systems” (26118508 to KN), “Integrative Understanding of Biological Phenomena with Temperature as a Key Theme” (15H05932 to KN), “Neuronal Diversity and Neocortical Organization” (25123709 to HH), “Adaptive Circuit Shift” (15H01430 to HH), and “Resonance Bio” (16H01426 to HH).

## Conflict of Interest Statement

The authors declare that the research was conducted in the absence of any commercial or financial relationships that could be construed as a potential conflict of interest.

## Footnotes

^1^http://imagej.nih.gov/ij
