# Supplementary material for: Differential Inputs to the Perisomatic and Distal-Dendritic Compartments of VIP-Positive Neurons in Layer 2/3 of the Mouse Barrel Cortex
Source: Front Neuroanat. 2016 Dec 20;10:124. doi: 10.3389/fnana.2016.00124 (PMC5167764; doi:10.3389/fnana.2016.00124)
Supplement: Supplementary file 1 [file DataSheet_1.docx]

Supplementary Material

Differential Inputs to the Perisomatic and Distal-Dendritic Compartments of VIP-Positive Neurons in Layer 2/3 of the Mouse Barrel Cortex

Jaerin Sohn, Shinichiro Okamoto, Naoya Kataoka, Takeshi Kaneko, Kazuhiro Nakamura, Hiroyuki Hioki

*** Correspondence:** Hiroyuki Hioki: hioki@mbs.med.kyoto-u.ac.jp

**Contents**

**1. Supplementary Figures**

Supplementary Figure 1. Estimation of the dendritic surface area.

Supplementary Figure 2. Dendritic morphology of VIP+ neurons located at the border of L1 and L2.

**2. Supplementary Tables**

Supplementary Table 1. A comparison of calculation methods for dendritic surface area.

Supplementary Table 2. The number of inputs per dendritic length.

## Supplementary Figures


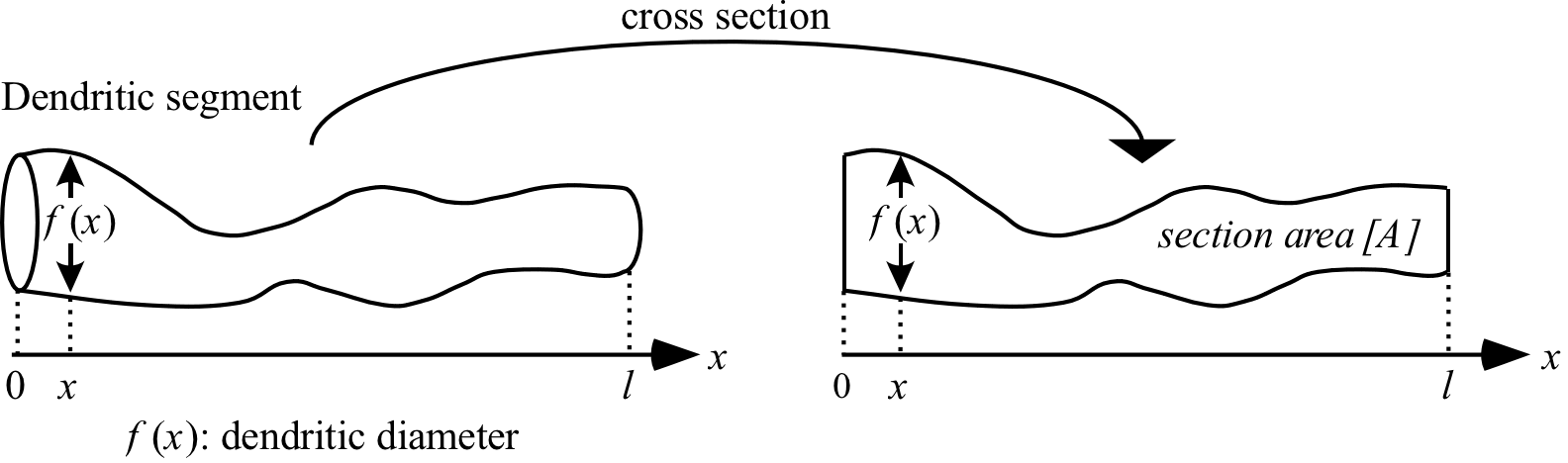


**Supplementary Figure 1. Estimation of the dendritic surface area.** The surface area of each dendritic segment can be calculated by the following formula:

$$\boldsymbol{Dendritic surface area}\left[ \boldsymbol{S}_{\boldsymbol{d}} \right]\boldsymbol{=}\int_{\boldsymbol{0}}^{\boldsymbol{l}} \boldsymbol{\pi f}\left( \boldsymbol{x} \right)\boldsymbol{dx}\boldsymbol{= \pi}\int_{\boldsymbol{0}}^{\boldsymbol{l}} \boldsymbol{f}\left( \boldsymbol{x} \right)\boldsymbol{dx}$$

The value *l* is the length of the dendritic segment of interest. The cross-section area can be obtained by the following formula:

$$\boldsymbol{Section area}\left[ \boldsymbol{A} \right]\boldsymbol{=}\int_{\boldsymbol{0}}^{\boldsymbol{l}} \boldsymbol{f}\left( \boldsymbol{x} \right)\boldsymbol{dx}$$

Therefore, the dendritic surface area of each segment can be calculated by the following formula:

$$\boldsymbol{Dendritic surface area}\left[ \boldsymbol{S}_{\boldsymbol{d}} \right]\boldsymbol{=\pi\times Section area [A]}$$

In the present study, to estimate the dendritic surface area of each segment, we first measured the cross-section area [*A*] by using the LSM 5 Image Examiner software. We then obtained the dendritic surface area [*Sd*] by multiplying π and [*A*], which is equivalent to the calculation of the integration of function *f*(*x*).


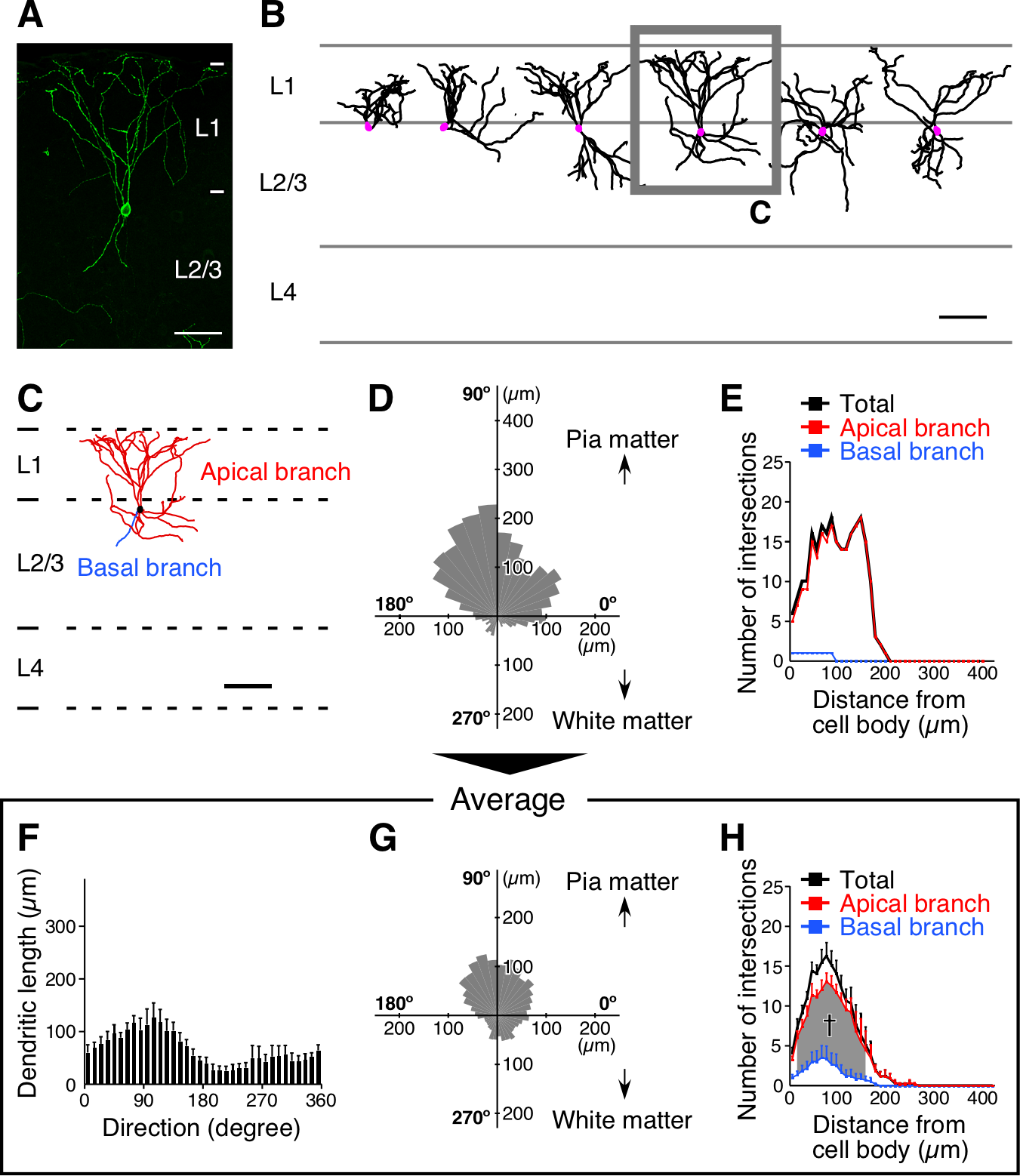


**Supplementary Figure 2. Dendritic morphology of VIP+ neurons located at the border of L1 and L2.** **(A)** Visualization of a VIP+ neuron at the border of L1 and L2. The 3D image stack of a 40-µm-thick coronal section was projected in the Z direction. Scale bar = 50 µm. **(B)** Reconstruction of six VIP+ neurons just beneath L1. The six VIP+ neurons with somata located at the border of L1 and L2 were manually reconstructed with NeuroLucida. The six VIP+ neurons had abundant apical dendritic branches especially in L1. Scale bar = 100 µm. **(C)** Reconstruction image of the VIP+ neuron in A and in the rectangle in B. We defined primary dendrites with at least one branch extending in L1 as apical branches (red) and those oriented in other directions as basal branches (blue). The basal branch was poorly developed. Scale bar = 100 µm. **(D)** Polar histogram of the VIP+ neuron dendrites depicted in C. The total dendritic length in each 10º is summed as a pie-shaped wedge. Dendrites toward the pia matter are shown at 90º, while those toward the white matter are shown at 270º. This polar histogram does not show bipolarity in the vertical direction. **(E)** Sholl analysis of the VIP+ neuron dendrites depicted in C. The number of dendrite intersections against the radial distance from the soma (every 10 µm) is plotted. Apical and basal branches are separately shown in red and blue, respectively. **(F, G)** Mean length of each radial bin from polar histograms of the six VIP+ neurons in B. The polar histogram does not clearly display bidirectional branch extension. Error bars, ± SEM. **(H)** Average of Sholl analyses of the six VIP+ neurons in B. The six VIP+ neurons exhibited more abundantly ramified apical branches than basal branches at the proximal portion. Error bars, ± SEM. †*p* < 0.05 using two-way ANOVA followed by Bonferroni *post hoc* multiple comparison test.

## Supplementary Tables

**Supplementary Table 1. A comparison of calculation methods for dendritic surface area.** We selected 10 dendrites at each distance from the soma by using random number tables. After tracing the dendrites with NeuroLucida, the dendritic surface area was measured with NeuroExplorer. We compared the dendritic surface areas calculated from the sectional area or measured by NeuroLucida and NeuroExplorer, and there were no significant differences in the values of dendritic surface areas (two-sided Student’s *t*-test).

| Distance from soma | Calculated from the section area (**A**) (µm^2^) | Measured by NeuroLucida (**B**) (µm^2^) | **A** / **B** | *p* value |
| --- | --- | --- | --- | --- |
| 20 µm | 67.0 ± 19.9 | 66.4 ± 19.2 | 1.01 ± 0.03 | 0.952 |
| 50 µm | 53.0 ± 20.0 | 53.1 ± 19.8 | 1.00 ± 0.03 | 0.995 |
| 100 µm | 48.2 ± 17.1 | 47.6 ± 17.4 | 1.01 ± 0.02 | 0.947 |
| 150 µm | 43.0 ± 12.7 | 42.4 ± 11.4 | 1.01 ± 0.07 | 0.906 |
| 200 µm | 55.7 ± 17.4 | 55.4 ± 18.1 | 1.01 ± 0.04 | 0.978 |
| 250 µm | 37.1 ± 5.1 | 36.7 ± 4.7 | 1.01 ± 0.02 | 0.878 |
| 300 µm | 41.0 ± 7.5 | 40.9 ± 6.8 | 1.00 ± 0.06 | 0.962 |

**Supplementary Table 2. The number of inputs per dendritic length.** At each dendritic segment, we counted the number of inputs and calculated the number of inputs per dendritic length. Data are given as mean ± SD.

| **VGluT1 Input** | | |
| --- | --- | --- |
| Distance from soma | Counted number of inputs | Number of inputs per dendritic length (10^-2^/µm) |
| 20 µm | 4.88 ± 3.20 | 22.6 ± 14.3 |
| 50 µm | 5.41 ± 1.94 | 24.9 ± 8.9 |
| 100 µm | 7.12 ± 2.99 | 34.6 ± 14.6 |
| 150 µm | 6.44 ± 2.78 | 30.4 ± 13.3 |
| 200 µm | 7.23 ± 2.78 | 32.4 ± 12.4 |
| 250 µm | 7.25 ± 2.54 | 33.5 ± 11.6 |
| 300 µm | 6.40 ± 3.38 | 30.5 ± 16.8 |

| **VGluT2 Input** | | |
| --- | --- | --- |
| Distance from soma | Counted number of inputs | Number of inputs per dendritic length (10^-2^/µm) |
| 20 µm | 2.67 ± 1.83 | 12.0 ± 8.4 |
| 50 µm | 2.50 ± 1.38 | 10.7 ± 5.9 |
| 100 µm | 3.11 ± 1.73 | 13.5 ± 7.9 |
| 150 µm | 3.41 ± 2.68 | 15.5 ± 12.5 |
| 200 µm | 2.56 ± 1.22 | 11.8 ± 5.9 |
| 250 µm | 2.75 ± 1.53 | 12.3 ± 7.3 |
| 300 µm | 4.67 ± 2.92 | 22.0 ± 14.5 |

| **VGAT Input** | | |
| --- | --- | --- |
| Distance from soma | Counted number of inputs | Number of inputs per dendritic length (10^-2^/µm) |
| 20 µm | 9.60 ± 4.43 | 45.2 ± 19.6 |
| 50 µm | 8.40 ± 3.51 | 37.7 ± 13.8 |
| 100 µm | 6.35 ± 2.74 | 29.5 ± 14.7 |
| 150 µm | 6.56 ± 2.32 | 30.2 ± 11.8 |
| 200 µm | 5.33 ± 1.97 | 25.5 ± 10.4 |
| 250 µm | 6.70 ± 1.95 | 32.0 ± 9.8 |
| 300 µm | 8.86 ± 3.18 | 38.3 ± 13.0 |

| **PV Input** | | |
| --- | --- | --- |
| Distance from soma | Counted number of inputs | Number of inputs per dendritic length (10^-2^/µm) |
| 20 µm | 6.33 ± 3.40 | 27.8 ± 14.2 |
| 50 µm | 4.27 ± 2.02 | 18.6 ± 8.4 |
| 100 µm | 2.13 ± 2.87 | 9.7 ± 12.7 |
| 150 µm | 1.27 ± 1.53 | 5.6 ± 6.3 |
| 200 µm | 0.45 ± 0.50 | 2.2 ± 2.2 |
| 250 µm | 0.14 ± 0.35 | 0.6 ± 1.6 |
| 300 µm | 0.25 ± 0.43 | 1.1 ± 2.0 |

| **SOM Input** | | |
| --- | --- | --- |
| Distance from soma | Counted number of inputs | Number of inputs per dendritic length (10^-2^/µm) |
| 20 µm | 1.78 ± 1.55 | 8.1 ± 6.9 |
| 50 µm | 1.39 ± 1.60 | 6.2 ± 6.9 |
| 100 µm | 1.39 ± 1.42 | 6.3 ± 6.4 |
| 150 µm | 2.29 ± 1.62 | 10.5 ± 7.1 |
| 200 µm | 2.36 ± 1.43 | 11.4 ± 7.2 |
| 250 µm | 4.25 ± 2.33 | 20.6 ± 10.2 |
| 300 µm | 5.75 ± 2.59 | 27.0 ± 11.0 |

| **VIP Input** | | |
| --- | --- | --- |
| Distance from soma | Counted number of inputs | Number of inputs per dendritic length (10^-2^/µm) |
| 20 µm | 0.68 ± 0.92 | 3.3 ± 4.2 |
| 50 µm | 0.47 ± 0.68 | 2.3 ± 3.3 |
| 100 µm | 0.63 ± 1.04 | 3.0 ± 4.9 |
| 150 µm | 0.63 ± 0.78 | 2.9 ± 3.6 |
| 200 µm | 0.60 ± 0.61 | 2.8 ± 2.8 |
| 250 µm | 0.75 ± 1.01 | 3.2 ± 4.4 |
| 300 µm | 0.57 ± 1.05 | 2.6 ± 4.5 |
